# Supplementary material for: Predicting visual function by interpreting a neuronal wiring diagram
Source: Nature. 2024 Oct 2;634(8032):113–23. doi: 10.1038/s41586-024-07953-5 (PMC11446822; doi:10.1038/s41586-024-07953-5)
Supplement: Supplementary file 6 — CRF and ERF predictions for individual TmY4 and TmY9 cells. Analogous to Supplementary Data 3, but for TmY target types. Shown are the top four monosynaptic pathways, the strongest pathway passing through each of the top ten intermediary types (ranking from Extended Data Fig. 7), and the trisynaptic pathway Tm1–TmY–Dm3–TmY (see the section entitled Prediction of spatial normalization). [file 41586_2024_7953_MOESM6_ESM.zip › DataS4/TmY9qΓèÑ/720575940628658709.pdf]

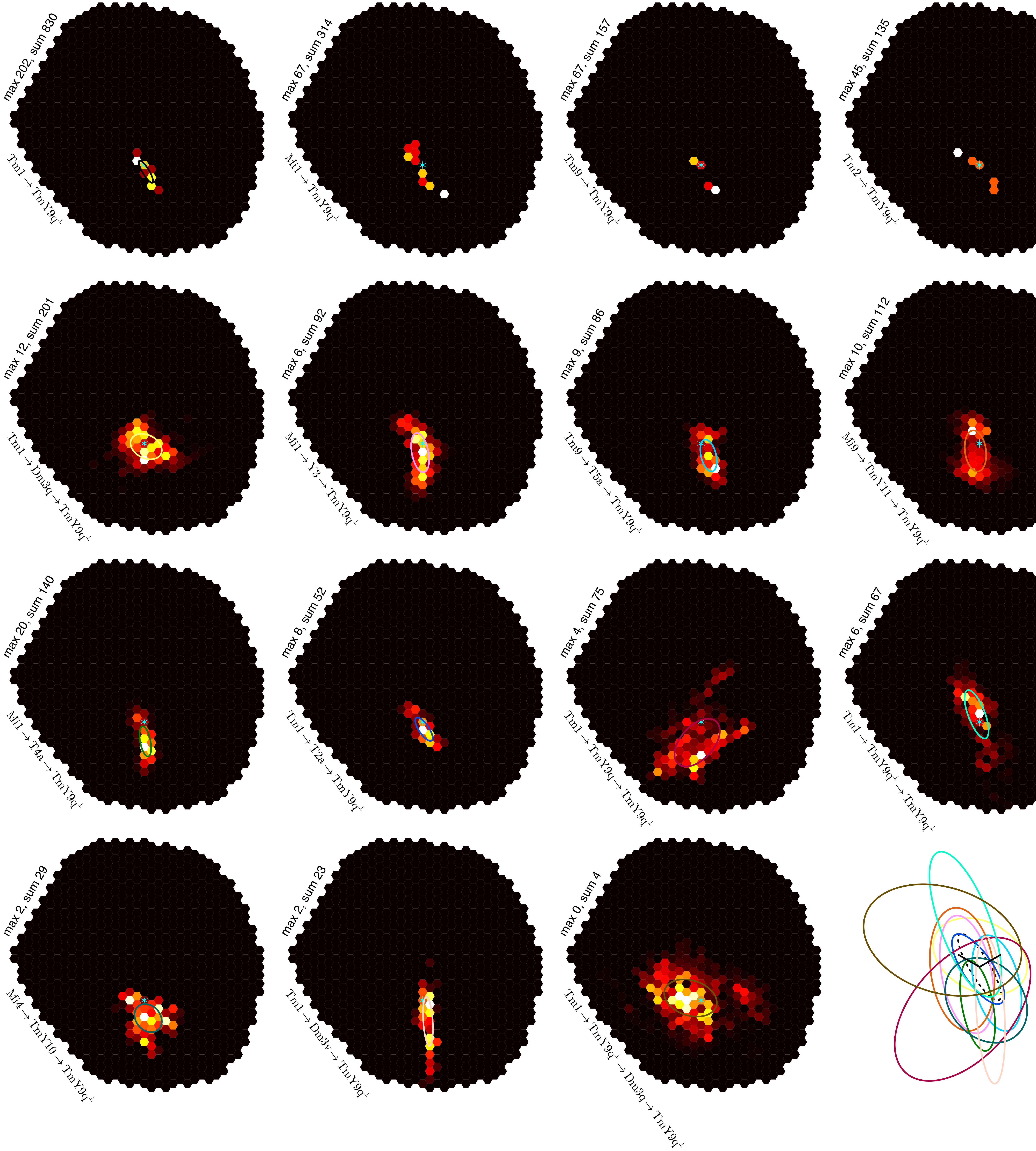
max 202, sum 830
Tm1 → TmY9q⊥
max 67, sum 314
Mi1 → TmY9q⊥
max 67, sum 157
Tm9 → TmY9q⊥
max 45, sum 135
Tm2 → TmY9q⊥
max 12, sum 201
Tm1 → Dm3q → TmY9q⊥
max 6, sum 92
Mi1 → Y3 → TmY9q⊥
max 9, sum 86
Tm9 → T5a → TmY9q⊥
max 10, sum 112
Mi9 → TmY11 → TmY9q⊥
max 20, sum 140
Mi1 → T4a → TmY9q⊥
max 8, sum 52
Tm1 → T2a → TmY9q⊥
max 4, sum 75
Tm1 → TmY9q⊥ → TmY9q⊥
max 6, sum 67
Tm1 → TmY9q⊥ → TmY9q⊥
max 2, sum 29
Mi4 → TmY10 → TmY9q⊥
max 2, sum 23
Tm1 → Dm3v → TmY9q⊥
max 0, sum 4
Tm1 → TmY9q⊥ → Dm3q → TmY9q⊥
